# Supplementary material for: Multi-scale and multi-context interpretable mapping of cell states across heterogeneous spatial samples
Source: Nat Commun. 2025 Aug 21;16:7814. doi: 10.1038/s41467-025-62782-y (PMC12370880; doi:10.1038/s41467-025-62782-y)
Supplement: Supplementary file 1 — Supplementary Information [file 41467_2025_62782_MOESM1_ESM.pdf]

Supplementary figures for:

Multi-scale and multi-context interpretable mapping of cell states across heterogenous spatial samples.

Author List

Patrick C.N. Martin[1], Wenqi Wang[2], Hyobin Kim[1], Henrietta Holze[2], Paul B. Fisher[3, 4, 5, 6], Arturo P. Saavedra[3, 7], Robert Winn[3, 4, 8], Esha Madan[3, 4, 5, 6], Rajan Gogna[3, 4, 5, 6, @], Kyoung Jae Won[1, @]

Affiliations

1- Department of Computational Biomedicine, Cedars-Sinai Medical Center, Hollywood, CA, USA.
2- Biotech Research and Innovation Centre (BRIC), University of Copenhagen, Copenhagen, Denmark.
3- Department of Surgery, Virginia Commonwealth University School of Medicine, 1200 E Broad St., P.O. Box 980011, Richmond, VA 23219, USA.
4- Massey Comprehensive Cancer Center, Virginia Commonwealth University, Richmond, VA, 23298, USA.
5- Division of Pulmonary Disease and Critical Care Medicine, Department of Internal Medicine, VCU School of Medicine, Virginia Commonwealth University, Richmond, VA, 23298, USA.
6- VCU Institute of Molecular Medicine, Department of Human and Molecular Genetics, Virginia Commonwealth University, School of Medicine, Richmond, VA, 23298, USA.
7- Department of Human and Molecular Genetics, Virginia Commonwealth University, School of Medicine, Richmond, VA, USA.
8- Department of Dermatology, VCU School of Medicine, Virginia Commonwealth University, Richmond, VA, 23298, USA.

@ - These authors jointly supervised this work.

Correspondence to-

Dr. KJ Won

Department of Computational Biomedicine, Cedars-Sinai Medical Center, Melrose Ave, Suite 540, Hollywood, Los Angeles, CA 90069

Email: KyoungJae.Won@cshs.org

Phone: +1 909-222-3020

Dr. Rajan Gogna

Department of Human & Molecular Genetics, VCU Institute of Molecular Medicine (VIMM), VCU Massey Cancer Center, Virginia Commonwealth University School of Medicine, Molecular Medicine Research Building (MMRB), 1220 East Broad Street, Richmond, VA 23298-0033

Email: rajangogna@gmail.com; rajan.gogna@vcuhealth.org

Phone: +1 6282526501

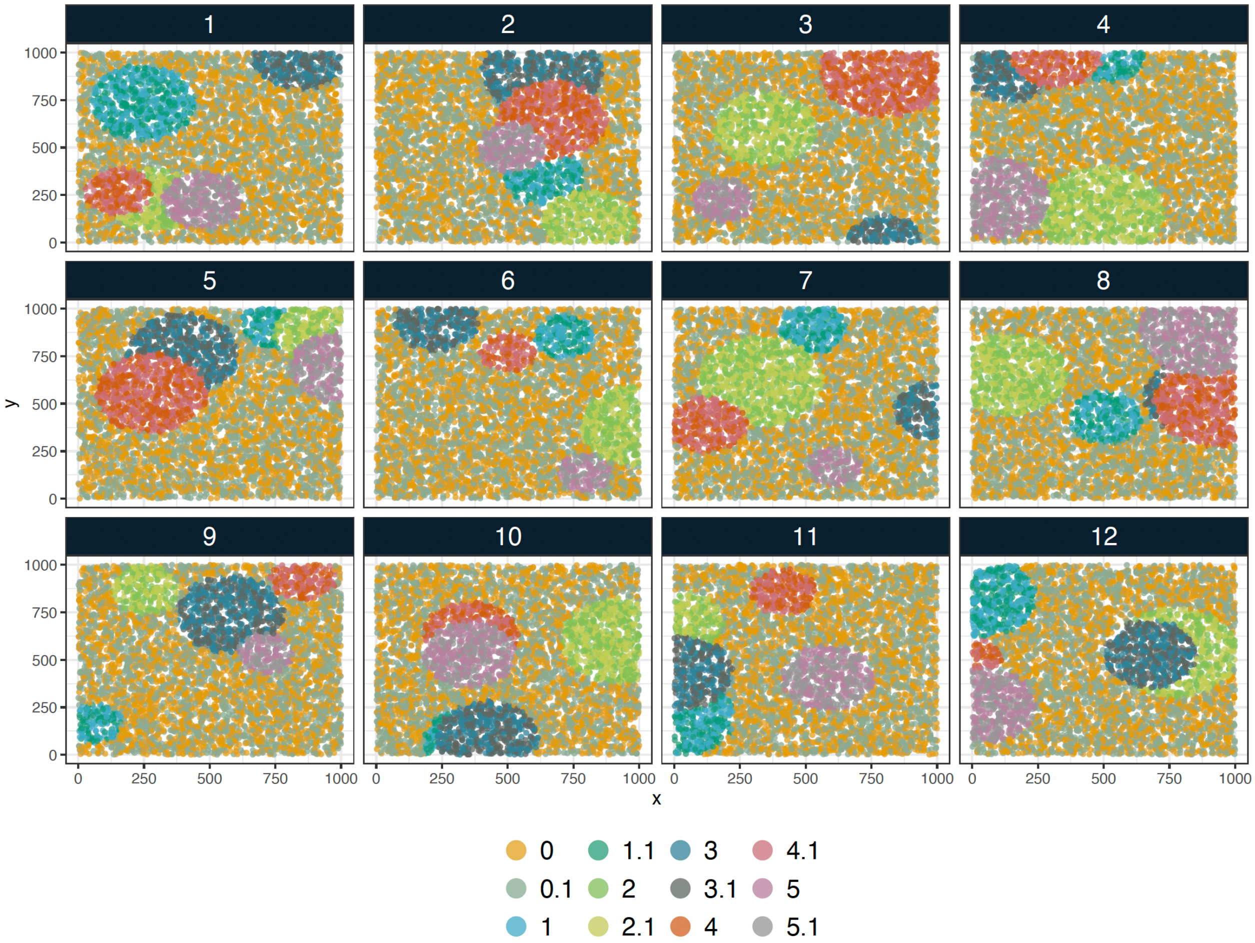

Figure S1: Synthetic spatial data – Circle regime. We produced 12 circle regime samples. The circle regime is characterized by a background territory in which 5 randomly sized and randomly placed (with overlaps) circle territories. Each territory (circle and background) can contain 2 cell types. The probability of differential gene expression between cell types is 0.5. Source data are provided as a Source Data file.

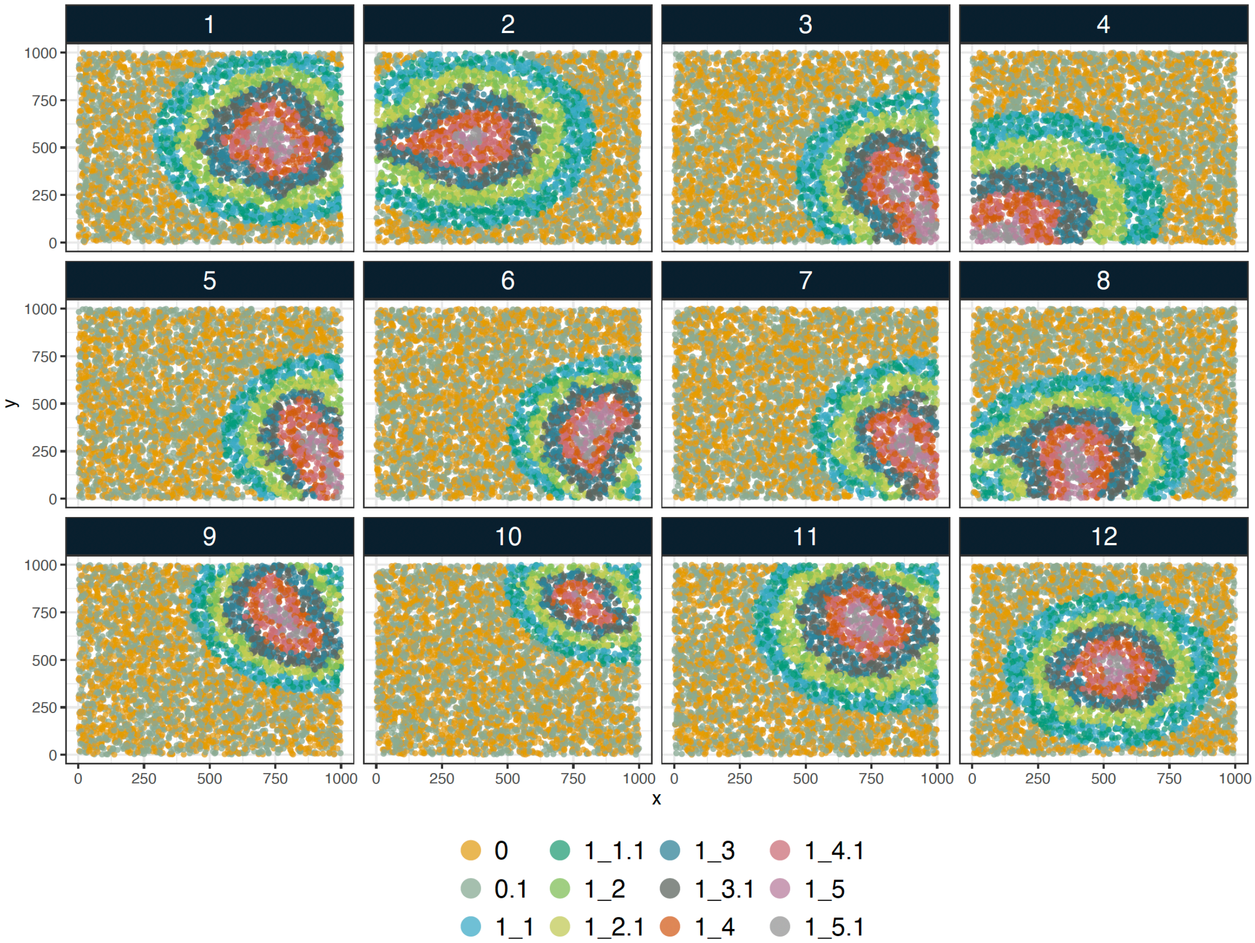

Figure S2: Synthetic spatial data – Layered regime. We produced 12 layered regime samples. The layered regime is characterized by a background territory in which a randomly sized and randomly placed circular territory. This circular territory is subdivided into layers. Each layer and the background can contain 2 cell types. The probability of differential gene expression between cells of the background and cell in the layer is 0.5. The probability of differential gene expression between cell in the layers in 0.05. Source data are provided as a Source Data file.

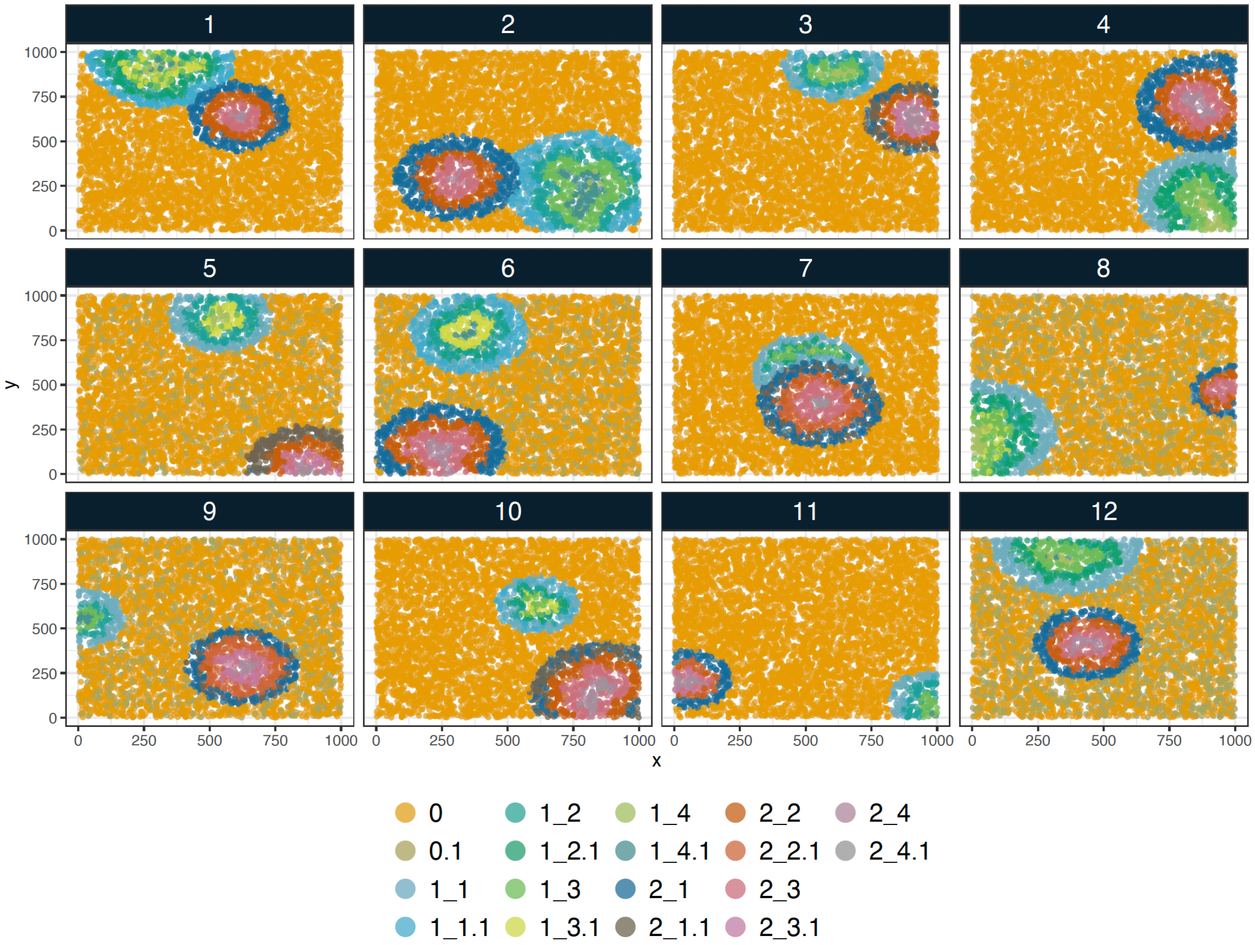

Figure S3: Synthetic spatial data – dropped regime. We produced 12 dropped regime samples. The dropped regime is characterized by a background territory in which 2 randomly sized and randomly placed circular territories. Each circular territory is subdivided into layers. Each layer and the background can contain either 1 or 2 cell types. The probability of differential gene expression between cells of the background and cell in the layer is 0.5. The probability of differential gene expression between cell in the layers in 0.05. Source data are provided as a Source Data file.

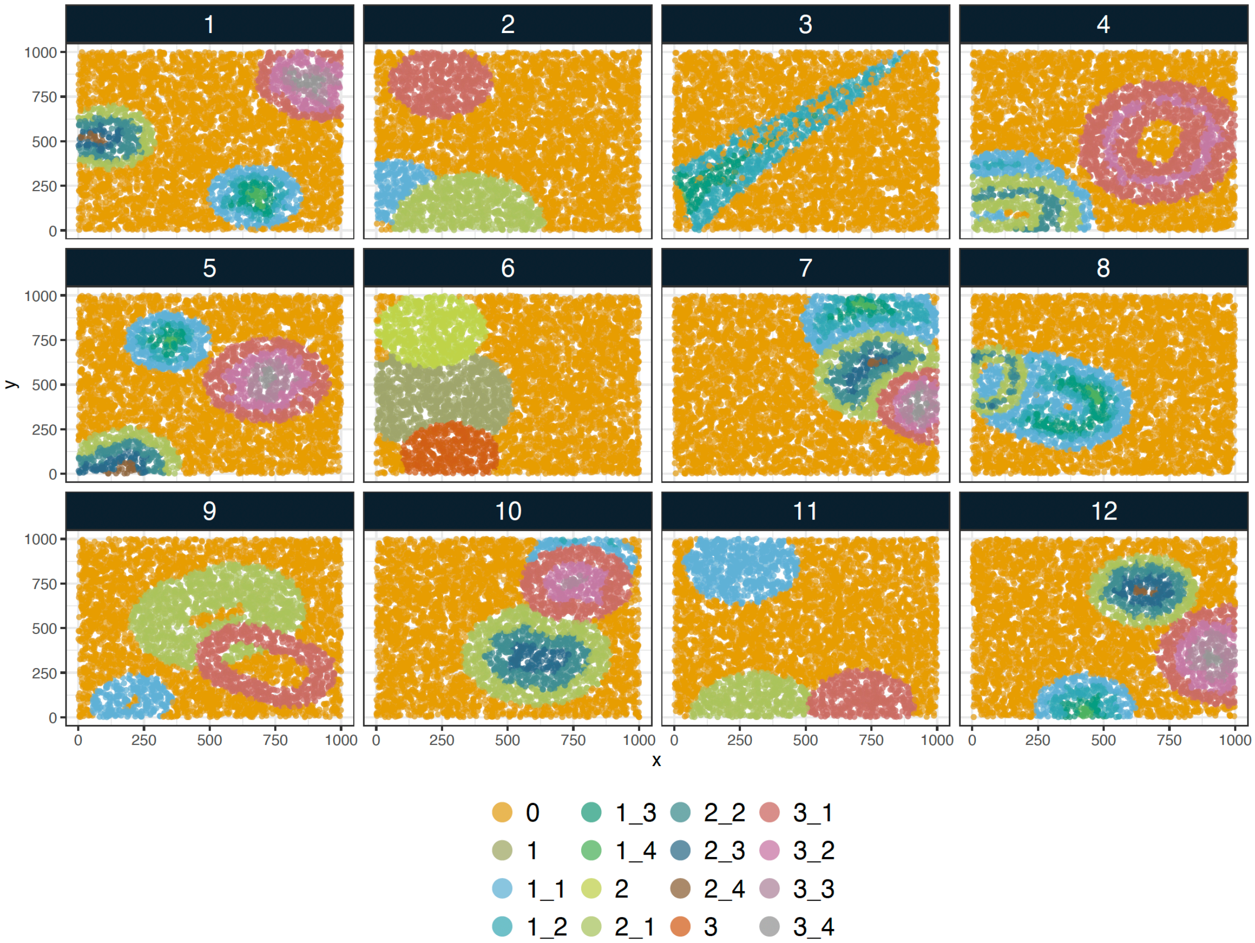

Figure S4: Synthetic spatial data – random one cell regime. We produced 12 of the random one cell regime samples. This regime is characterized by random territory types (circle, rod, or chaos maps), of random size and with random number of layers. Each territory of layer will contain only one cell type. The probability of differential gene expression between cells of the background and cell in the layer is 0.5. The probability of differential gene expression between cell in the layers in 0.05. Source data are provided as a Source Data file.

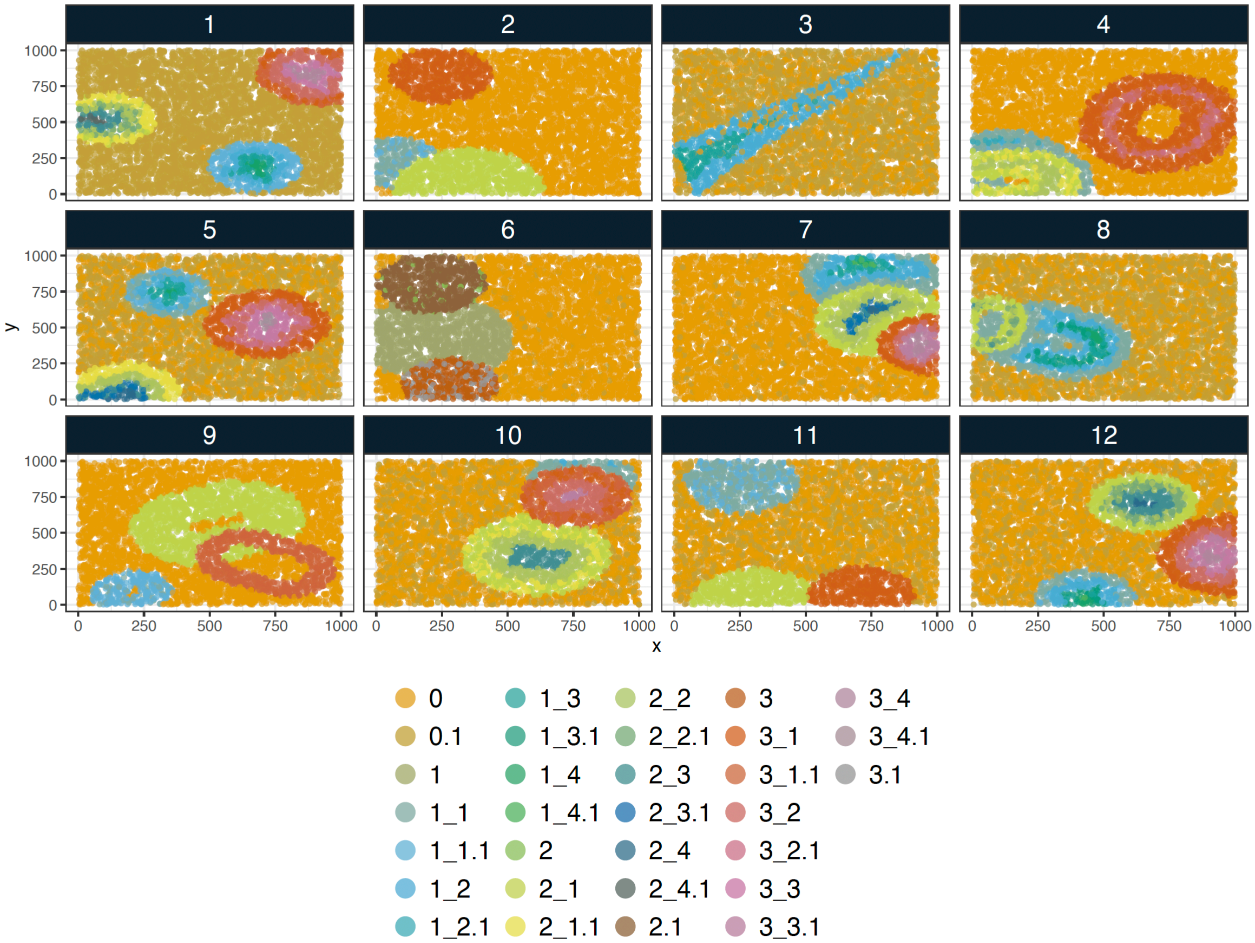

Figure S5: Synthetic spatial data – random two cell regime. We produced 12 of the random two cell regime samples. This regime is characterized by random territory types (circle, rod, or chaos maps), of random size and with random number of layers. Each territory of layer will contain two cell types. The probability of differential gene expression between cells of the background and cell in the layer is 0.5. The probability of differential gene expression between cell in the layers in 0.05. Source data are provided as a Source Data file.

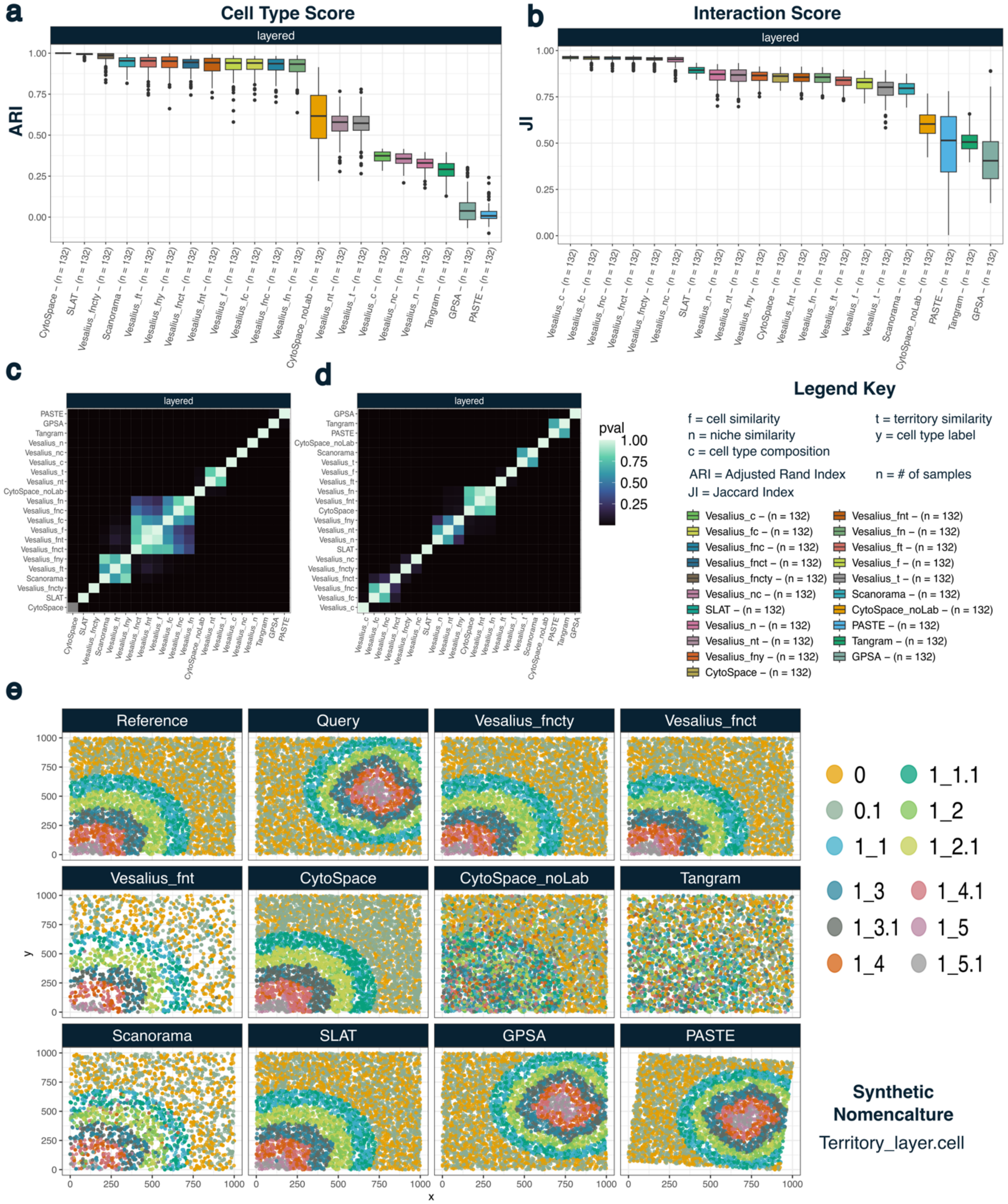

Figure S6: Benchmarking mapping performance in synthetic spatial data – layered regime. We provide a Legend Key on the figure describing the nomenclature of Vesalius cost matrices as well as the meaning of acronyms. **(a)** Performance comparison in recovering cell types across samples using an ARI in synthetic

spatial data. We compared Vesalius using 14 different cost matrices to SLAT, CytoSpace, CytoSpace with no cell type labels (noLab), Tangram, Scanorama, PASTE, and GPSA. Number of samples (*n*) used in each box plots included in axis labels. The box shows the interquartile range (IQR = Q3 − Q1), with a line at the median, and whiskers extending to the most extreme values within 1.5×IQR. **(b)** JI of cell interactions. To account for the spatial context of cells, we computed the JI between cell type labels in the neighborhood of mapped cells (k=6). **(c)** Heat map representing p-values after performing a two-side Wilcoxon rank sum test between tools on ARI scores. **(d)** Heat map representing p-values after performing Wilcoxon rank sum test between tools on JI scores. **(e)** Example mapping event in the layered regime. We show mapping events for 3 Vesalius cost matrices (*fncty*, *fnct*, *fnt*). Source data are provided as a Source Data file.

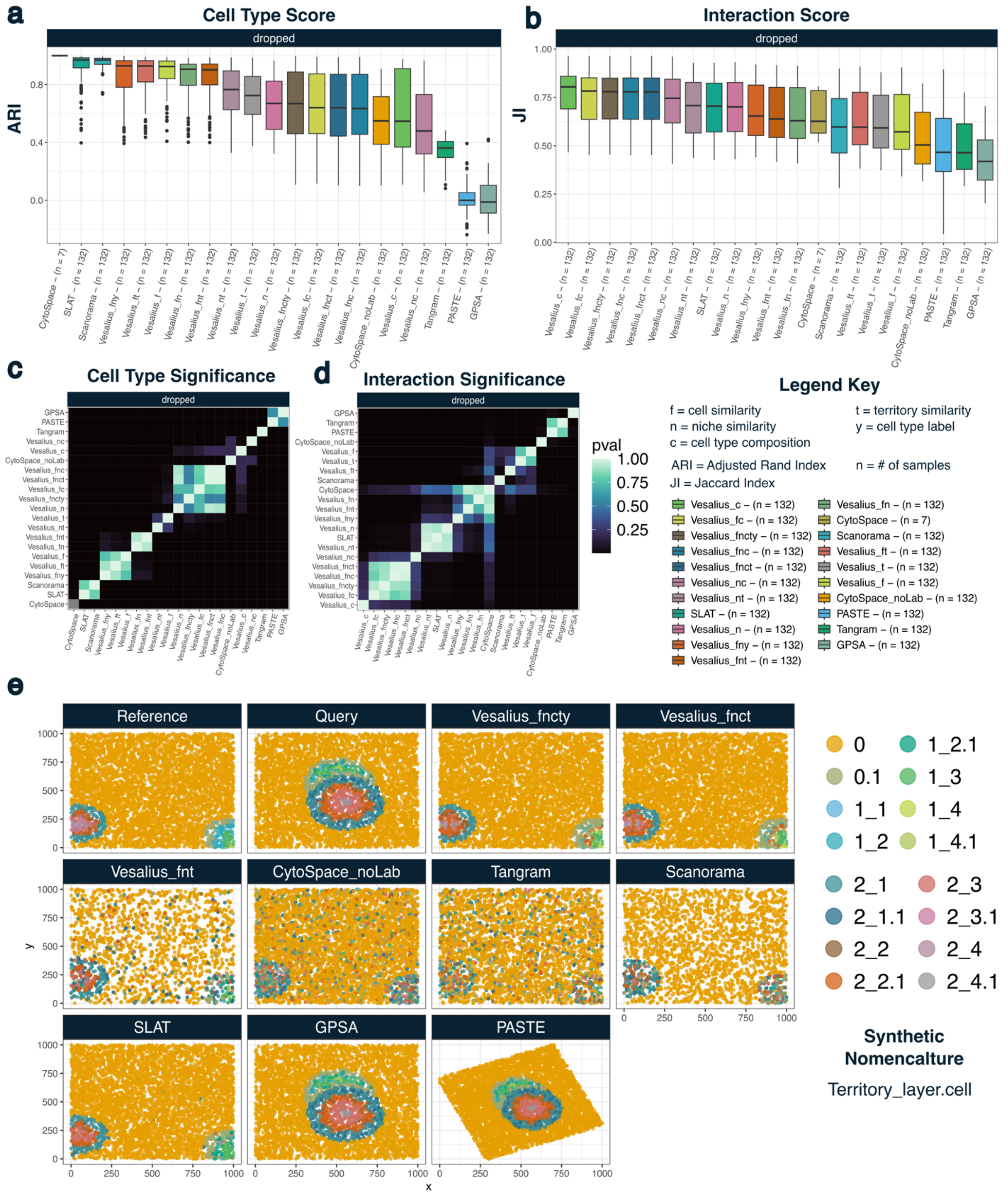

Figure S7: Benchmarking mapping performance in synthetic spatial data – dropped regime. We provide a Legend Key on the figure describing the nomenclature of Vesalius cost matrices as well as the meaning of acronyms.

(a) Performance comparison in recovering cell types across samples using an ARI in synthetic spatial data. We compared Vesalius using 14 different cost matrices to SLAT, CytoSpace, CytoSpace with no cell type labels (noLab), Tangram, Scanorama, PASTE, and GPSA. Number of samples (*n*) used in each box plots included in axis labels. The box shows the interquartile range (IQR = Q3 − Q1), with a line at the median, and whiskers extending to the most extreme values within 1.5×IQR. **(b)** JI of cell interactions. To account for the spatial context of cells, we computed the JI between cell type labels in the neighborhood of mapped cells (k=6). **(c)** Heat map representing p-values after performing a two-side Wilcoxon rank sum test between tools on ARI scores. **(d)** Heat map representing p-values after performing a two-side Wilcoxon rank sum test between tools on JI scores. **(e)** Example mapping event in the layered regime. We show mapping events for 3 Vesalius cost matrices (*fncty*, *fnct*, *fnt*). Source data are provided as a Source Data file.

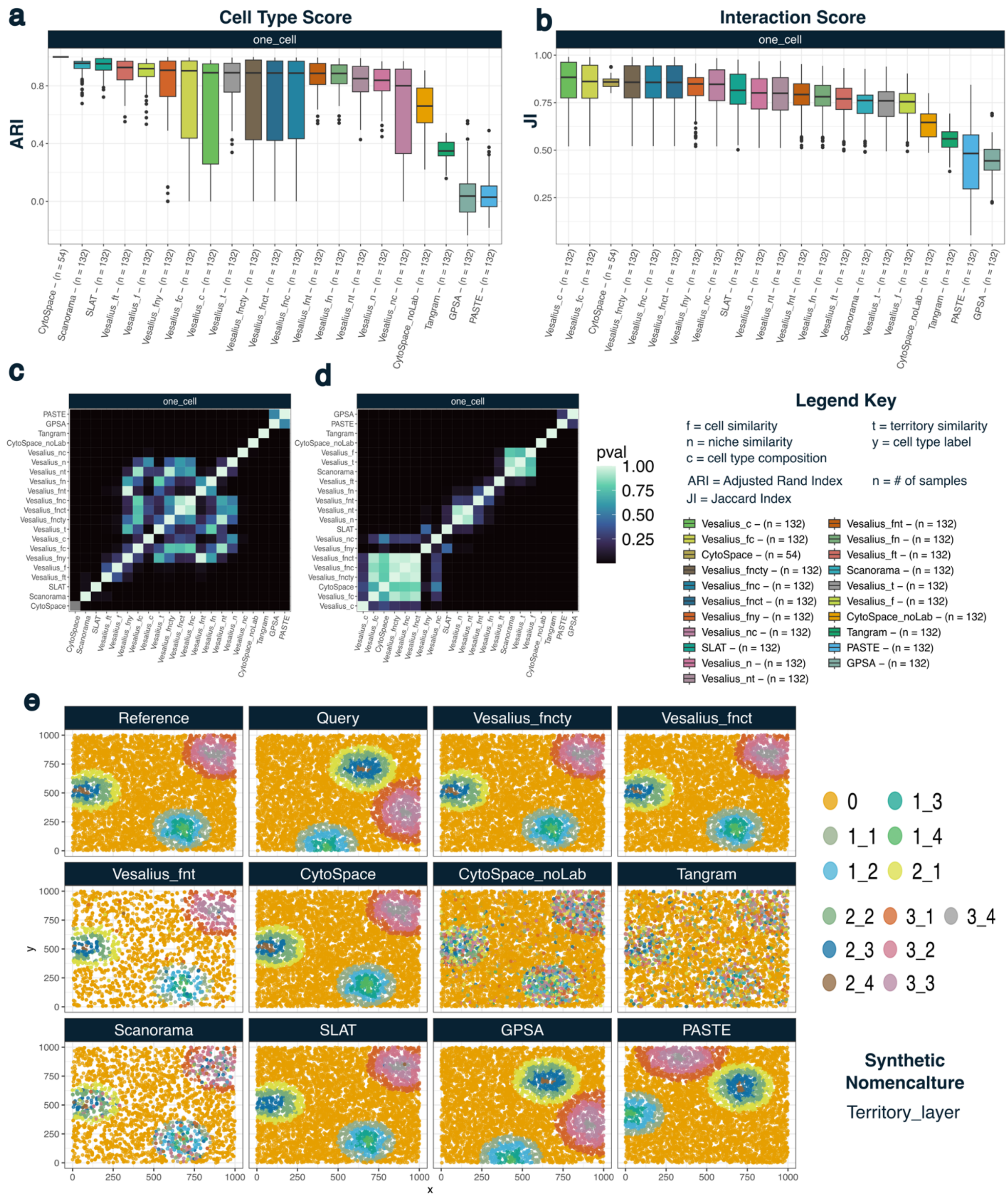

Figure S8: Benchmarking mapping performance in synthetic spatial data – random one cell regime. We provide a Legend Key on the figure describing the nomenclature of Vesalius cost matrices as well as the meaning of acronyms. **(a)** Performance comparison in recovering cell types across samples using an ARI in synthetic spatial data. We compared Vesalius using 14 different cost matrices to SLAT, CytoSpace, CytoSpace with no cell type labels (noLab), Tangram, Scanorama, PASTE, and GPSA. Number of samples (*n*) used in each box plots included in axis labels. The box shows the interquartile range (IQR = Q3 − Q1),

with a line at the median, and whiskers extending to the most extreme values within 1.5×IQR. **(b)** JI of cell interactions. To account for the spatial context of cells, we computed the JI between cell type labels in the neighborhood of mapped cells (k=6). **(c)** Heat map representing p-values after performing a two-side Wilcoxon rank sum test between tools on ARI scores. **(d)** Heat map representing p-values after performing a two-side Wilcoxon rank sum test between tools on JI scores. **(e)** Example mapping event in the layered regime. We show mapping events for 3 Vesalius cost matrices (*fncty*, *fnct*, *fnt*). Source data are provided as a Source Data file.

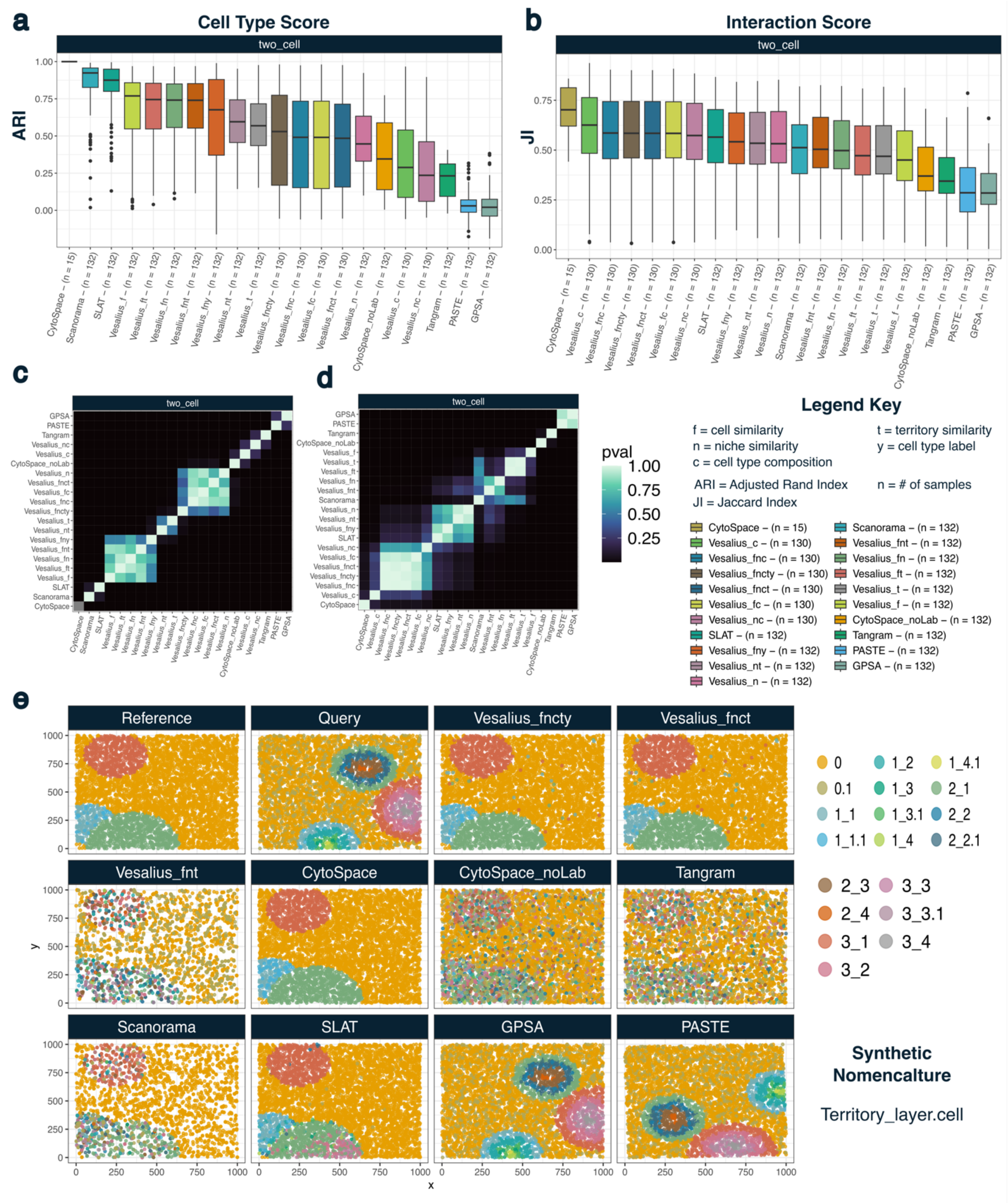

Figure S9: Benchmarking mapping performance in synthetic spatial data – random two cell regime. We provide a Legend Key on the figure describing the nomenclature of Vesalius cost matrices as well as the

meaning of acronyms. **(a)** Performance comparison in recovering cell types across samples using an ARI in synthetic spatial data. We compared Vesalius using 14 different cost matrices to SLAT, CytoSpace, CytoSpace with no cell type labels (noLab), Tangram, Scanorama, PASTE, and GPSA. Number of samples (*n*) used in each box plots included in axis labels. The box shows the interquartile range (IQR = Q3 − Q1), with a line at the median, and whiskers extending to the most extreme values within 1.5×IQR. **(b)** JI of cell interactions. To account for the spatial context of cells, we computed the JI between cell type labels in the neighborhood of mapped cells (k=6). **(c)** Heat map representing p-values after performing a two-side Wilcoxon rank sum test between tools on ARI scores. **(d)** Heat map representing p-values after performing a two-side Wilcoxon rank sum test between tools on JI scores. **(e)** Example mapping event in the layered regime. We show mapping events for 3 Vesalius cost matrices (*fncty*, *fnct*, *fnt*). Source data are provided as a Source Data file.

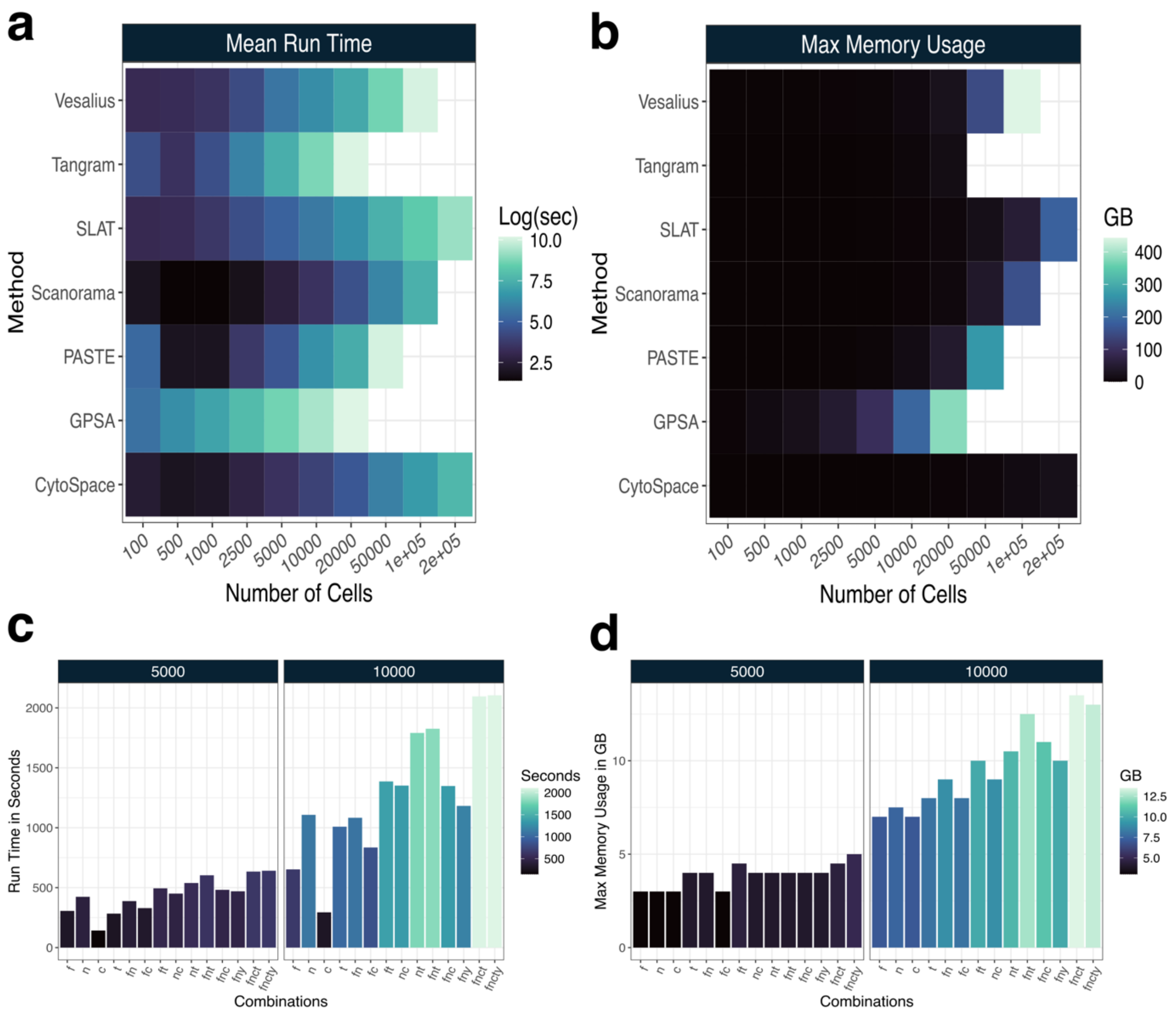

Figure-S10: Mean computational performance across 3 runs. Each missing tile signifies that a run exceeds either max run time (12 hours) or max allocated memory (480GB). **(a)** Mean Run time across all tools with increasing number of cells **(b)** Mean peak memory usage across all tools with increasing number of cells. **(c)** Mean Run time across Vesalius cost matrix combinations for 5000 and 1000 cells. **(d)** Mean peak memory usage across Vesalius cost matrix combinations for 5000 and 1000 cells. Source data are provided as a Source Data file.

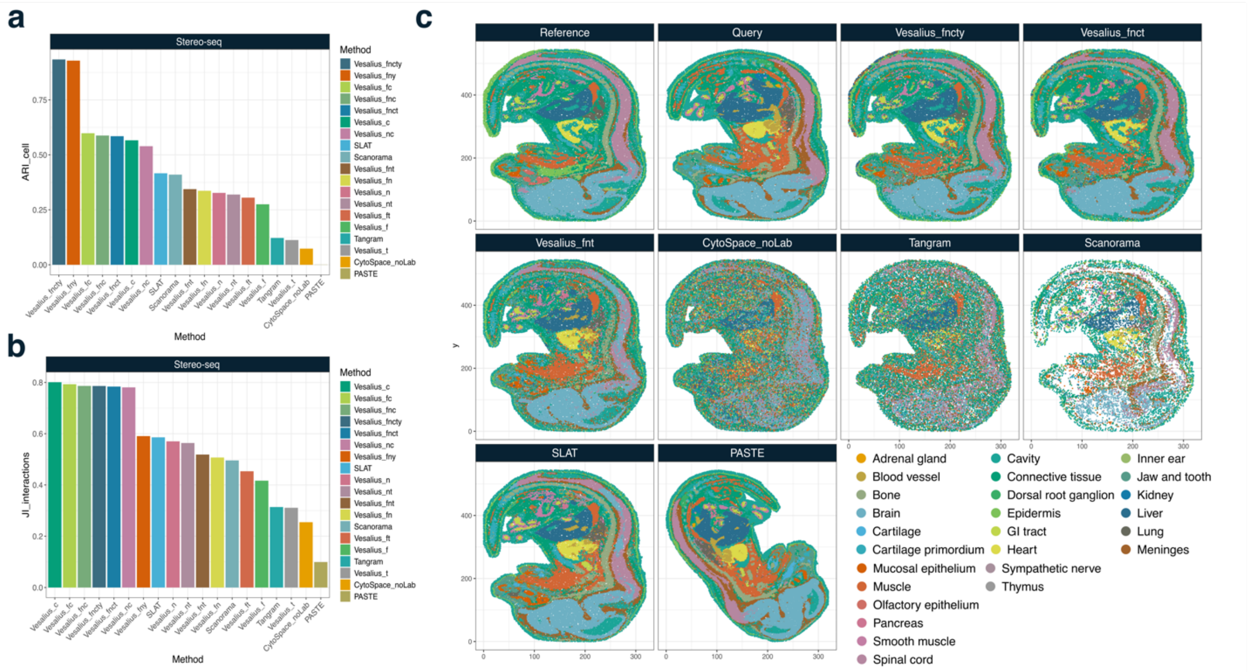

Figure S11: Benchmarking mapping performance in Stereo-seq mouse embryo. (a) ARI of mapped cell type labels between a reference Stereo-seq mouse embryo data set and a query Stereo-seq mouse embryo data set. We compared Vesalius using 14 different cost matrices to SLAT, CytoSpace, CytoSpace with no cell type labels (noLab), Tangram, Scanorama, and PASTE. CytoSpace failed to map cells from query to the reference. We still show the performance of CytoSpace when no labels are provided. Adding cell type labels or composition of cellular neighborhoods improves Vesalius's ability to accurately map cells across samples even outperforming SLAT and Scanorama. **(b)** JI of cell interactions. **(c)** Example mapping event in Stereo-seq mouse embryo. Source data are provided as a Source Data file.

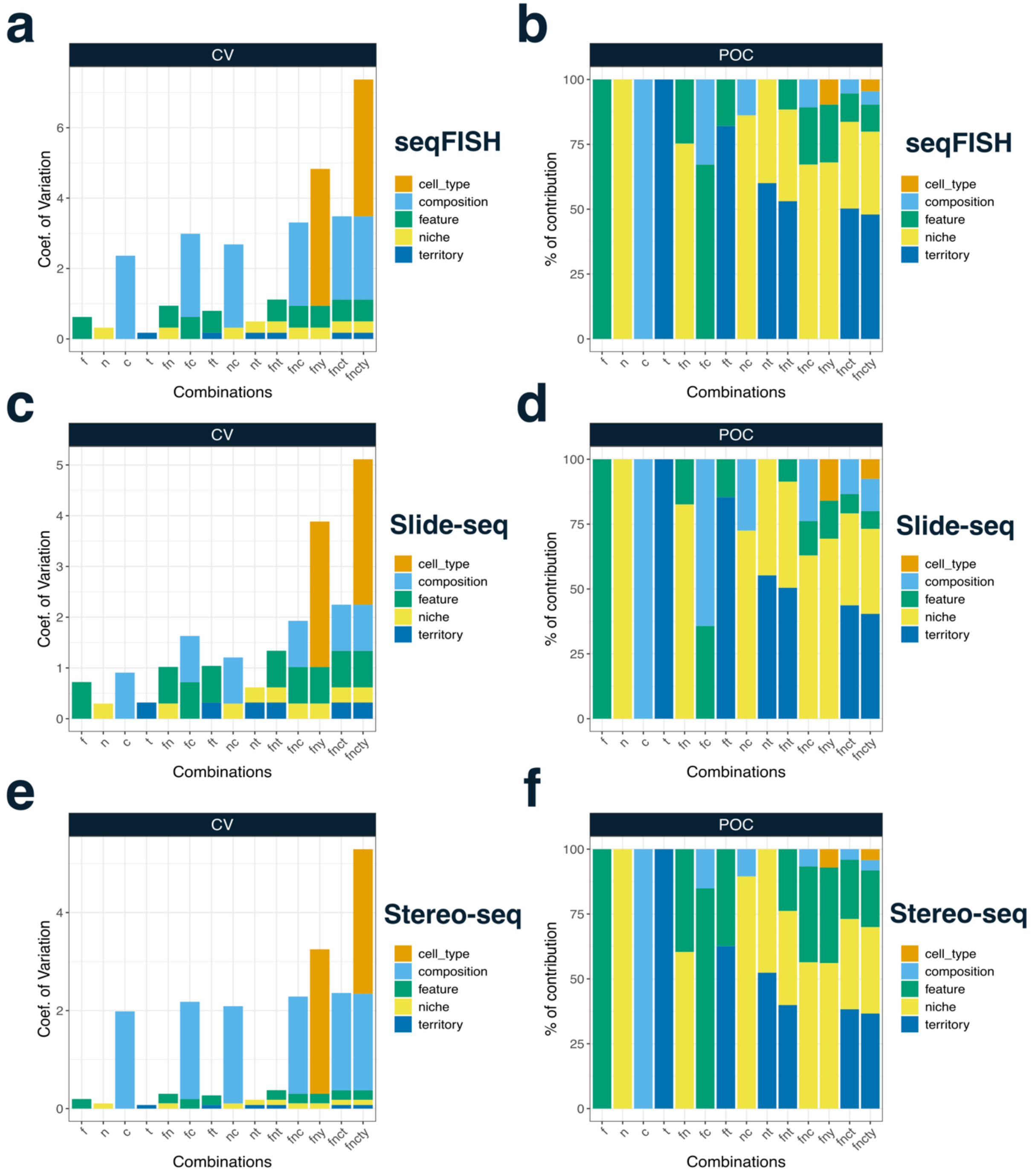

Figure S12: Cost matrix combinations and their contribution to mapping. We compared the contribution of different cost matrices in 3 biological spatial transcriptomics data sets using CV and POC. **(a)** CV for each combination in seqFISH. The higher the score the more this cost matrix is able to discriminate between cells during mapping. **(b)** POC in seqFISH shows how much each cost matrix contributes to total cost. The higher the contribution the higher the correlation/Jaccard indices were. **(c)** CV for each combination in Slide-seq V2. **(d)** POC in Slide-seq V2 **(e)** CV for each combination in Stereo-seq. **(f)** POC in Stereo-seq . Source data are provided as a Source Data file.

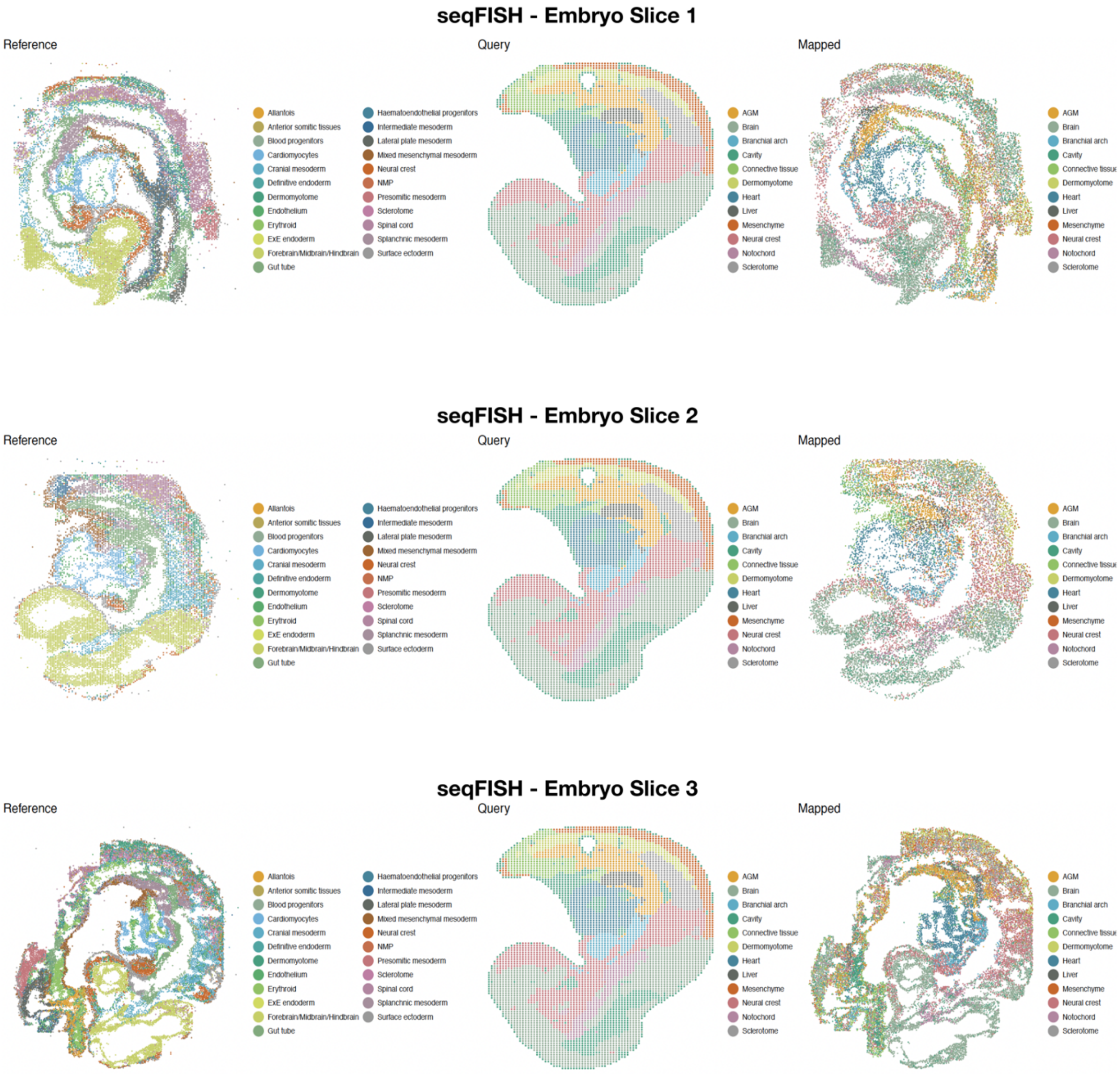

Figure-S13: Mapping of Stereo-seq onto all 3 seqFISH Mouse embryo sections. We mapped the Stereo-seq mouse embryo (Query – middle) onto 3 seqFISH mouse embryo slices (Reference - left) taken from the same data set. The mapping results (Mapped – right) show a good concordance with the expected position of cells in the reference data set. Source data are provided as a Source Data file.

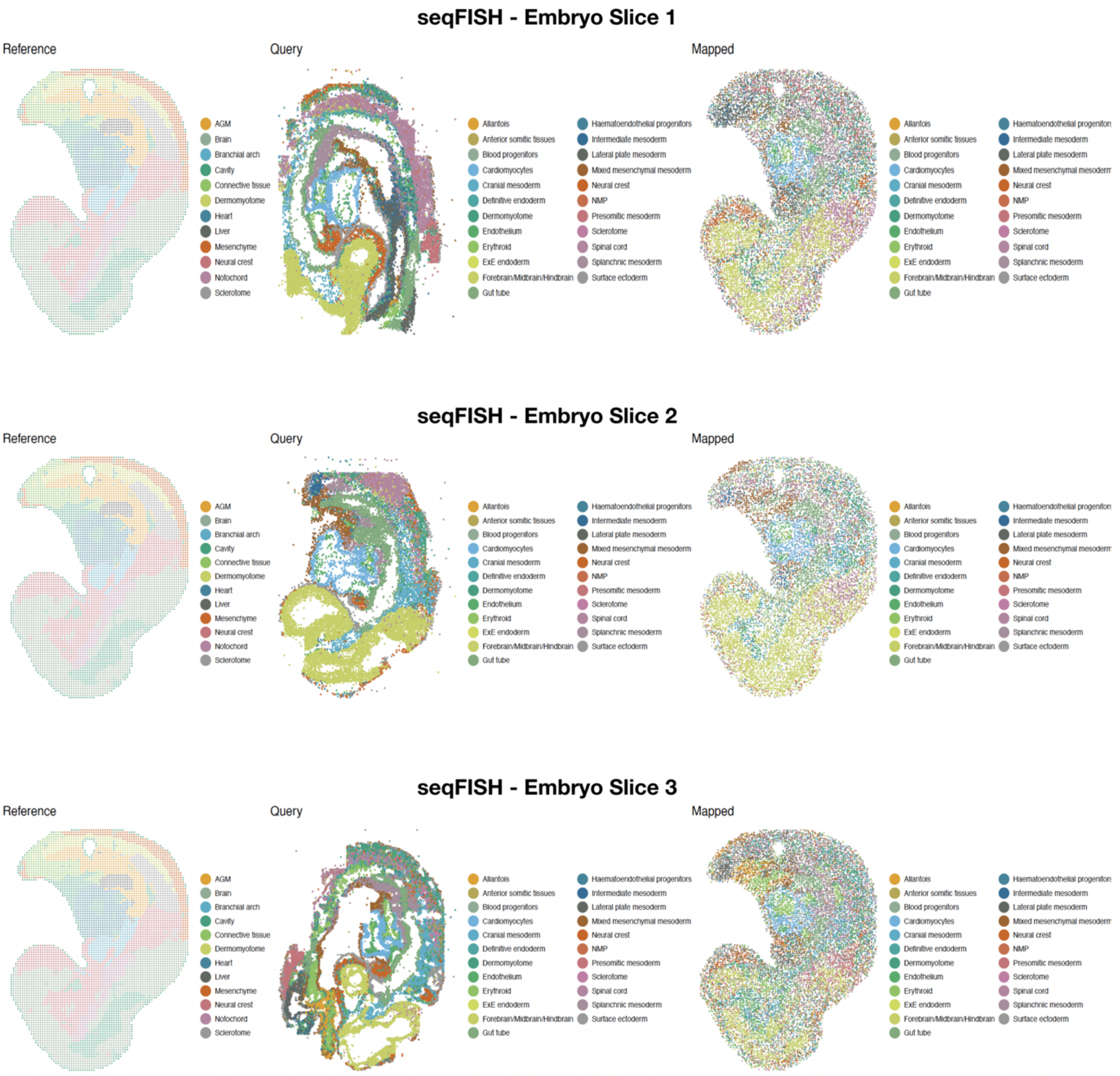

Figure-S14: Mapping of seqFISH data onto Stereo-seq data. We applied our mapping strategy to map seqFISH mouse embryo (Query – middle) onto Stereo-seq mouse embryo (Reference – left). The mapping results show that we can recover expected tissue structures despite different cell type label representations. Source data are provided as a Source Data file.

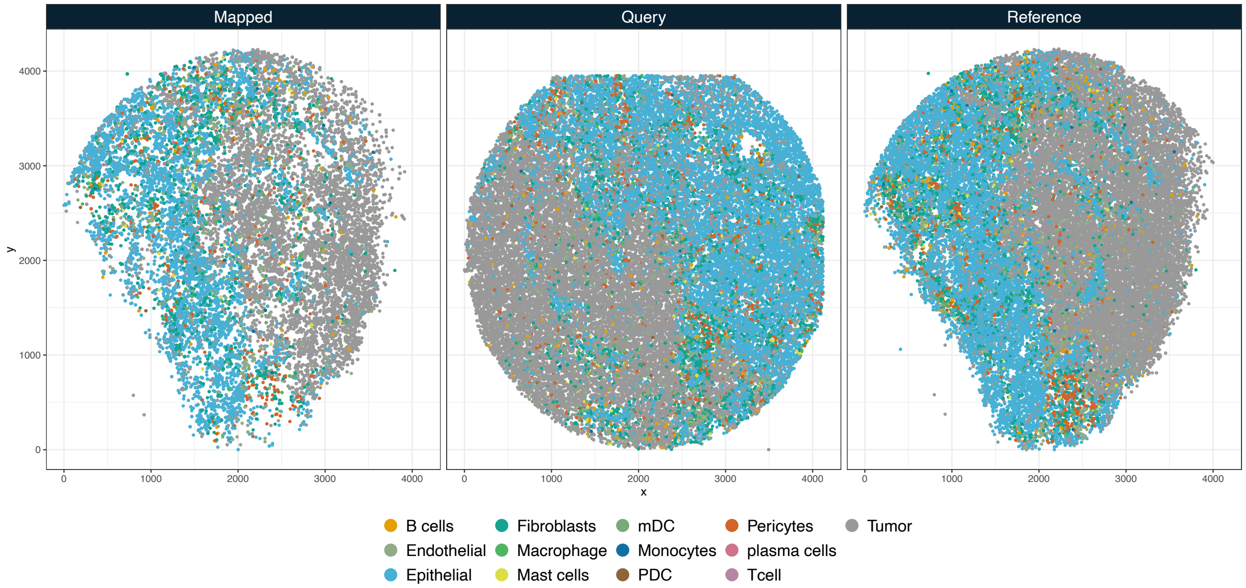

Figure-S15: Mapping of cells across prostate cancer Slide-seq V2 data. We mapped cells from the query data set onto the reference. Mapped cells are shown in the mapped panel. The mapping results indicate that Vesalius recovers the location of cells as well as the location of the tumor. Source data are provided as a Source Data file.

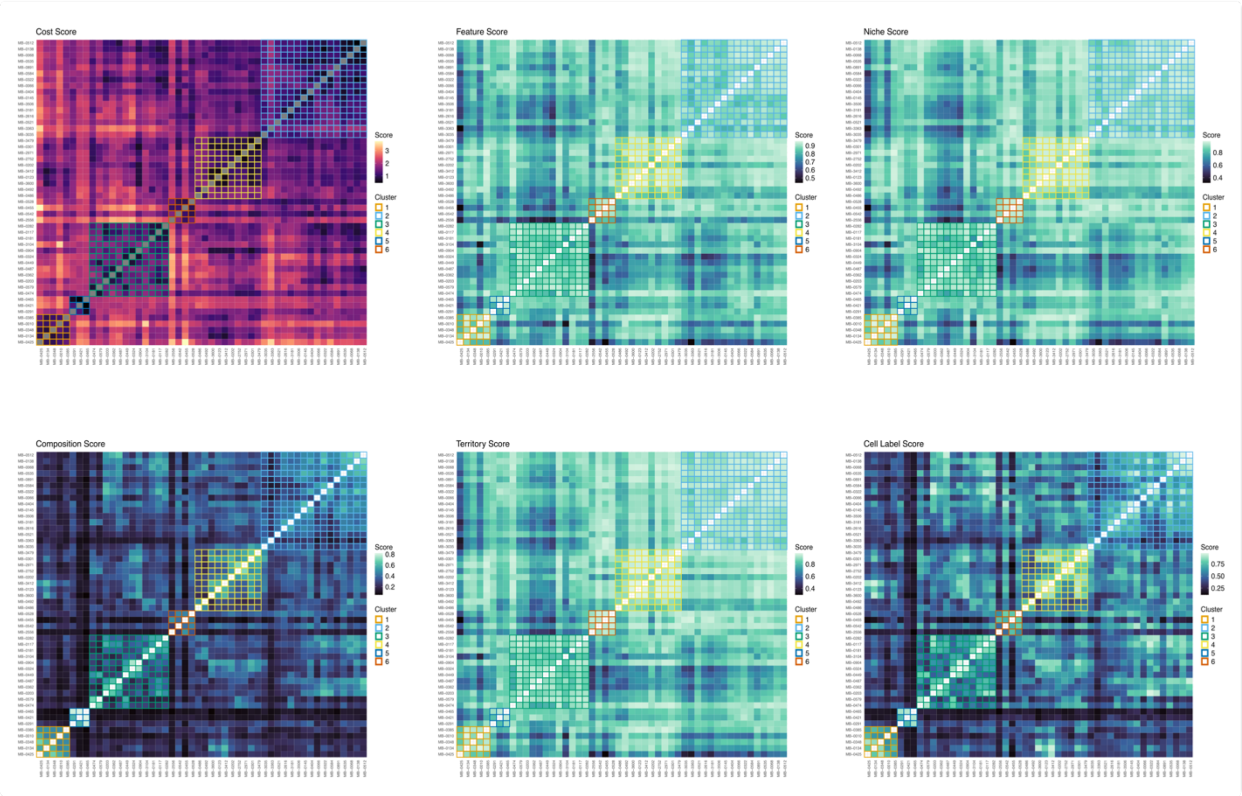

Figure-S16: ER positive patient clustering. We clustered ER positive samples with respect to their overall mapping cost to each other samples. The cost is shown is the upper left followed by feature, niche, composition, territory, and cell label scores (from left to right – top to bottom). While less demarcated than in ER negative samples, we see that cluster 3 (green) for instance could be subdivided into smaller clusters driven by cell composition and cell label scores. This subdivision is not as demarcated in continuous cell state metrics such as feature, niche, and territory. These results suggest a dissociation between discrete cell types assigned to cells and the cell state continuum cells find themselves. Source data are provided as a Source Data file.

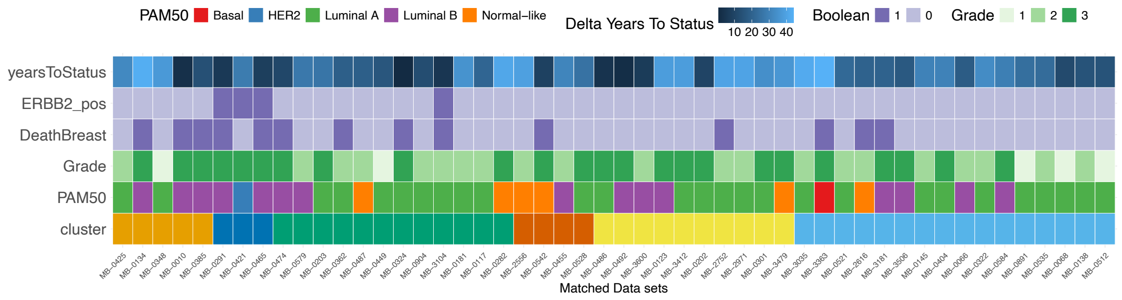

Figure S17: Clinical metrics associated with the clustering of ER positive patients.
We use Boolean to represent True (1) and False (0) values. Using total mapping cost as clustering distance, we clustered patients based on multi-scale spatial similarity and compared them with patient level metrics (PAM50, Years to status, ERBB2 positive, death status). For instance, cluster 3 (green) exhibits a majority of the Luminal-A cancer subtype. Overall, our clustering results show that patient status can be at least partially encoded in a cancer's multi-scale organization. Source data are provided as a Source Data file.
