# Supplementary material for: Multi-scale and multi-context interpretable mapping of cell states across heterogeneous spatial samples
Source: Nat Commun. 2025 Aug 21;16:7814. doi: 10.1038/s41467-025-62782-y (PMC12370880; doi:10.1038/s41467-025-62782-y)
Supplement: Supplementary file 2 — Description of Additional Supplementary Files [file 41467_2025_62782_MOESM2_ESM.pdf]

Description of Additional Supplementary Files:

Supplementary Data 1: DEGs during Axolotl brain regeneration (differential gene expression across time at the zone of injury)

Supplementary Data 2: Inter cluster DEGs in mouse embryo (differential gene expression between brain clusters after mapping)

Supplementary Data 3: Intra cluster DEGs in mouse embryo (differential gene expression across time in the same cluster)

Supplementary Data 4: Clustering and labelling of IMC patients in Figure 6 and Supplementary Figures S15 and S16
